# Implementation of contingency management in probation agencies using a case controlled longitudinal design: a PDSA study protocol

**DOI:** 10.1186/2194-7899-1-7

**Published:** 2013-12-19

**Authors:** Faye S Taxman, Danielle S Rudes

**Affiliations:** Department of Criminology, Law & Society, George Mason University, New York, USA

## Abstract

**Background:**

Moving evidence-based practices (EBPs) from clinical research settings to real world work environments is challenging. Grounded in the work of Proctor and colleagues on “bench-trench” partnerships, quality improvement studies use a bench (academic)-trench (practitioner) model that incorporates “practice knowledge” while building a feedback process throughout the various change phases (Social Work Research, 27, 67-69). Yet, few implementation studies give adequate attention to longitudinal collection of key organizational and individual level-information.

**Method/Design:**

The Justice Steps (JSTEPS) project uses a PDSA (Plan-Do-Study-Act) quasi-experimental design that combines active and engaged researcher and practitioner learning collaborations about EBP adoption and implementation. The academic researchers and practitioners (Federal probation and judicial staff) work together to implement contingency management (CM) into routine practice. Each site participates in two learning collaborative meetings, receives six feedback reports, and accesses telephone consultation on the design and implementation of the CM procedure. The study protocol allows examination of the implementation of CM over 24 months in five settings. Each setting proceeds based on the sites’ own pace of adopting CM. This study uses a case controlled pre-post design to measure individual level data and a pre-post design for staff level outcomes. The outcomes of interest are reduced substance use and increased time on probation. The project data collection includes individual-level client data and organizational and staff data to assess the implementation of the CM in five probation agencies through three periods: inception, implementation and sustainability. Qualitative methods include observations and interviews combined with data gathering during learning sessions. Individual level client data includes attendance, status at required events, and arrests.

**Discussion:**

This project contributes to the current understanding of how contextual factors affect implementation decisions. The protocol allows each site to develop their own tailored CM protocol and a process for implementing CM, compatible with the local socio-political environment. Feedback loops are important for fostering attention to CM implementation issues.

**Electronic supplementary material:**

The online version of this article (doi:10.1186/2194-7899-1-7) contains supplementary material, which is available to authorized users.

## Background

Contingency management (CM) is an evidence-based practice (EBP) (National Institute on Drug Abuse 2010) that theoretically appears compatible with the basic strategies used by judicial or probation officials within the U.S. criminal justice system. The justice system routinely uses reinforcers to address compliance with behavior for desired drug- and crime-free behaviors. The compatibility of positive reinforcers with the existing system lies in the similarity between the core concepts of CM and the principles of effective punishment: swift, certain, and increasingly intensified responses. Given this consistency with the core functions of justice processing, CM implementation in justice systems should be relatively easy to implement (Rogers 2003).

Contingency management has wide applications in the area of behavior change. In substance abuse treatment settings, CM interventions reduce drug use and increase treatment retention for a wide variety of drug abusers (Stitzer et al. 2010). Among the core components of any CM protocol is a focus on reducing or eliminating certain behavior(s) (e.g., abstinence from drug and alcohol use) and the use of structured and transparent rewards or incentives as the primary driver of behavior change. As such, CM protocols consistently use systems in which points are assigned to desired positive behaviors. Clients earn rewards via redeeming earned accumulated points. In prior work on implementing CM within drug and alcohol addiction treatment, clinicians established CM guidelines detailing which behavior(s) needed changing and how rewards could be earned. In such studies, both clients and clinicians understand that the rewards are achievable only with demonstrated positive behavior in line with the target goal (Stitzer et al. 2010).

The emphasis on rewards is unusual for justice settings. The justice system’s focus is on punishment as a tool to improve obedience to the law as well as probation supervision. The introduction of rewards as a tool to achieve compliance presents a challenge since the compatibility for the structure of response exists, but using this type of response is rare. Within the justice system, drug courts often co-mingle rewards and sanctions, with preference to sanctions (Taxman et al. 2007a; Marlowe et al. 2008; Rossman et al. 2011). The attention to sanctions over incentives is due to disapproval of incentives (as being too “soft”), the general lack of attention to the importance of varying degrees of support for short-term goal achievement, and the notion that people should not be rewarded for “doing what they should do.” For example, even though probation officers (POs) in drug courts regularly voice support for using incentives (Murphy et al. 2012), rewards generally conflict with the punishment-oriented culture of the justice system. The structure associated with CM, although similar to the recommended manner to use swift and certain sanctions, confronts the tendency for the legal system to defer to individualized decision-making (tailoring to the needs of an individual). Although offenders’ expected behaviors are clear, POs’ discretion means that responsive action is sporadic when offenders do not comply with expectations (Steiner et al. 2011). In fact, drug court judges generally do not respond to all of offenders’ negative behaviors (Rossman et al. 2011). This *non-response*, coupled with overwhelming cultural conflicts regarding incentives, implies that the justice system focuses little attention on specific behaviors. This creates a lack of consistency regarding expectations. Likewise, studies have also found that justice actors do not routinely use structured response schemes such as guidelines. The failure to do so may affect the deterrent ability of sanctions (Paternoster 1987). A swift, certain, and increasingly graduated set of responses are important for deterrence (Paternoster 1987; Taxman et al. 1999).

CM implementation is challenging in most settings, even those outside justice settings. This difficulty is especially visible within the U.S. substance abuse treatment system. For example, Ducharme and colleagues found that exposure to training and positive feedback from peer organizations regarding CM did not persuade clinics to use incentives such as motivational vouchers (Ducharme et al. 2007). Instead, clinical structural factors such as revenue sources, accreditation, and type of clinical programming had a more pronounced negative influence on the use of CM. This stands in contrast to clinics’ adoption of pharmacotherapies for addiction treatment, for which exposure to the clinical practice through participation in a research network was more influential on adoption decisions than the programs’ structural characteristics. Counselors, like POs within the justice system, are hesitant to use motivational incentives given the widespread belief that one should not pay for “compliance.” (Kirby et al. 2006) This cultural conflict occurs even with research studies suggesting that incentive strategies, such as the ability to draw a prize at random from a fishbowl or low-cost or symbolic rewards, increase compliance to drug treatment conditions without negative consequences (Petry & Bohn 2003).

### Significance

For the most part, scientists have designed and executed CM protocols as part of research studies. No study shows clinicians designing their own CM protocols and using them with their patients or clients. The existing literature focuses on the efficacy of CM in substance abuse and other settings, but studies have not examined *implementing* CM in justice settings. Adapting CM to the justice environment raises certain questions that are unanswerable within the existing research on CM, drug treatment courts, or other justice system innovations. These unanswered questions include: 1) Who should determine the amount and type of incentives and who should provide the incentives to offenders (e.g., judges, probation officers, defense attorneys, treatment providers)?; 2) Which and how many behaviors should be targeted?; 3) What reward schedule should be used to tally achievement of the various target behaviors?; 4) How frequently should points be provided and when should rewards be given?, and 5) How should sanctions or punishment coincide with CM protocols? The complexity of the justice system is unlike other behavioral health arenas in that the typical offender has a number of additional expectations not seen in substance abuse or other treatment programming, where most CM protocols are tested. While a range of behaviors are expected of offenders under supervision (e.g., being crime free, drug and/or alcohol free, employed or in school, in a stable [and drug-free] housing environment, attending treatment or probation sessions on time, abiding by curfews, no gun/weapon ownership/use) the sheer number and comprehensive nature of this range of target behaviors presents difficulties for CM implementation. In fact, the justice system expects pro-social behavior in all categories, making it more difficult to identify *key* desired behaviors (e.g., those worthy of rewards).

Few studies examine how these justice systems and their priorities affect EBP implementation. One way of considering the internal and external contingencies related to EBP implementation is discussed in the seminal piece on EBP transportability by Schoenwald and Hoagwood. The setting, population, staffing, external stakeholders, and other factors may affect the core components of EBPs(Schoenwald & Hoagwood 2001). Although not well understood, transportability issues have the potential to shape an intervention into a form that does not adhere to its essential core components, around which its evidence base was established.

One noted factor affecting the design and form of CM is the relationship between the probation office and the judiciary. Problem solving courts report using rewards as part of their foundation, although studies find that these courts prefer to use discretionary rewards. Additionally, these courts regularly use rewards for a wide range of behaviors such as drug free urines, drug treatment and court attendance. This means that the judiciary, and the associated organizational actors such as the prosecutor and/or defense attorney, may have different priorities regarding which behaviors to target and reward. They use different mechanisms to recognize individual progress. CM implies a more structured and consistent approach to dispensing rewards. When using structured rewards, accumulated points directly translate into having the opportunity to get a prize from the fishbowl, obtaining a financial or social incentive, or deferring to the next reward level through a universal process that all justice actors understand and use consistently.

Another justice-related issue is the mixing of sanctions and rewards. The traditional CM literature only focuses on positive reinforcers and does not look at the integration of positive and negative reinforcers within the same program. However, the justice system is required to sanction certain negative behaviors such as any criminal behavior that results in an arrest, weapon possession, or physical violence. CM protocols must be flexible in their implementation to allow each site to use appropriate procedures to address such negative behaviors in a predictable and timely fashion.

## Methods/Design

**Figure 1:**
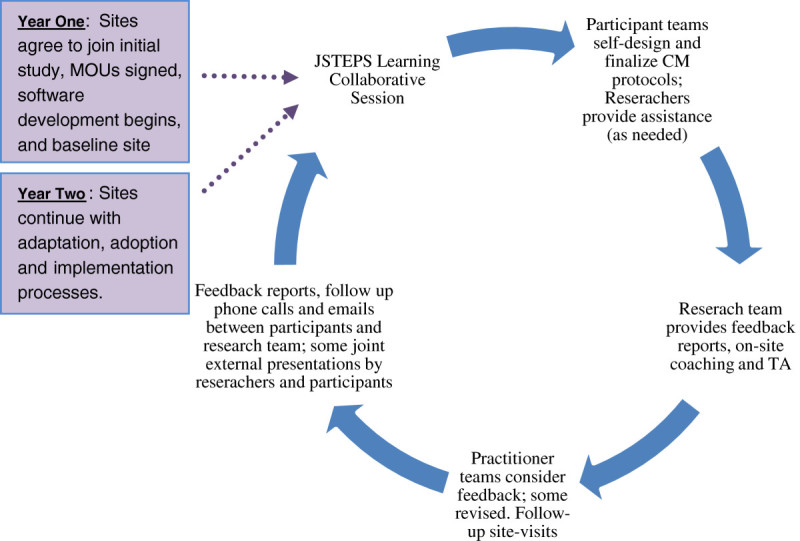
**The JSTEPS quant + qual study & data collection process.**

The JSTEPS project set out to assess CM implementation processes among community corrections actors using a PDSA (plan-do-study-act) quality improvement process (Deming 1982; Shewhart 1931). Within the PDSA process, the implementation and research phases of the project occur concurrently so that the project unfolds as both an implementation activity and a research study from inception. This is in contrast to studies where research on both process and outcome tends to follow various steps of implementation. The JSTEPS PDSA has four phases: 1) participating sites learn about CM as an EBP; 2) they design a CM protocol; 3) they refine the CM protocol based on initial outcomes, and 4) they assess the impact on their system. Along with two collaborative learning sessions where research sites learn about CM from a design and implementation perspective, the protocol includes technical assistance and quarterly feedback on how each site’s plan and self-designed point system align with the science underpinning contingency management. Sites then refine their protocols and implement JSTEPS in their agencies. To understand the impact of the PDSA process on implementation progress, the JSTEPS project uses an intensive, longitudinal mixed-method, collaborative research design that privileges feedback loops and includes surveys, interviews, observations, training and technical assistance (TA), coaching and continual feedback cycles. Figure 1 graphically depicts this process. Organizational surveys capture the attitudes and opinions of staff prior to the JSTEPS study and 12 months after beginning the use of CM and observational data via interviews and focus groups were used for up to 24 months after the study start date. Further data collection on the clients served by the system is included in the study design to assess whether the implemented CM affects offender outcomes.

The study was funded for two years by the National Institute on Drug Abuse as part of the Criminal Justice Drug Abuse Treatment Studies (CJ-DATS) cooperative and additional funds were provided by George Mason University to collect one year additional qualitative interviews and site visits. It was designed as a feasibility study of the concept of using CM in justice settings with an emphasis on the stages of adoption. For the system level measures, a pre-post organizational survey with longitudinal observations of system processes provided the system level variables on perceptions of rewarding in the sanctioning system, compatibility with the organizational goals and operations, and impact on outcomes such as completion of treatment and completion of probation. (A common outcome variable in justice-related research is the revocation or failure rates from probation due to non-compliance with program requirements. Completion of probation is a positive outcome.) At the individual level, a case control longitudinal design was used to identify whether or not the use of rewards affected the trajectory of probation and treatment outcomes and drug use positive rates. This design was selected due to the limited time frame of the study, the research questions, and the ability to use comparative sites (including the five study sites and five control sites) to assess client outcomes. The emphasis on individual level outcomes is important to demonstrate that system changes can affect individual level outcomes.

### Site selection and agreements

JSTEPS study sites include five U.S. Federal Probation offices in different U.S. districts. Although prior literature suggests that federal probation and district courts are committed to incorporating EBPs (Sherman 2009), JSTEPS ensures sites’ interest by choosing only sites that have adopted risk-screening tools for identifying offenders at the highest risk for violations of probation or parole (Taxman et al. 2007b). Part of the study assesses the decision processes sites encounter while considering CM adoption and implementation. Thus, sites are not required to *implement* CM after attending the first learning collaborative unless they decide it is in their best interest. However, this article focuses on the components of the study protocol that examine how and when sites implement CM after making that initial decision.

At the project’s initiation, JSTEPS researchers initiated Memoranda of Understanding (MOUs) with each site. The MOUs outline project goals and expectations regarding CM implementation. The JSTEPS team also submitted a study protocol with George Mason University’s Human Subjects Review Board (HSRB) and they approved the research. Table 1 presents an overview of the selected sites with data on each sites’ JSTEPS study participants, where and how they use CM and information about relevant contextual factors at each location.

**Table 1:** Study sites & pre-implementation reactions to CM (n = 39 site participants)*

Site characteristics	Site one	Site two	Site Three	Site four	Site Five
**Implementation setting**	Problem solving court	Problem solving court	Problem solving courts (2)	General probation	Halfway house
**Site participants involved**	Judge	Judge	Judge	POs	Judge
	AUSA	AUSA	AUSA		FPD
	FPD	FPD	FPD		POs
	POs	POs	POs		
	Treatment providers				
	n = 8	n = 5	n = 14	n = 5	n = 7
**Used some type of incentives before JSTEPS**	Yes	Yes	No	No	No
**Inter-organizational dynamics**	Have worked together for 4 years, know each other well, but maintain adversarial legal process, team makes decisions via consensus; team defers to judge even on minor decisions	Have worked together for 2 years, maintain traditional adversarial rules when talking through most issues, try to come to group consensus	Just establishing one court with a second less than one year old, very focused on team work and consensus decision-making	Probation has autonomy when implementing new programs; PO Chief works to maintain relationships with key leaders in other agencies in the system.	Probation has autonomy when implementing programs in halfway house, does not include other organizational actors in the process
**Initial acceptability of CM (within probation)**	Dedicate a supervising and frontline PO to court. Both POs receptive to incentives. Has PO using workbooks to facilitate offender change. Will use CM in court process.	Dedicate 1 PO to the court. PO has social work background and is very receptive to the idea of using incentives. PO uses workbooks to facilitate offender change. Will use CM in court process.	Dedicate 2 POs to court; both have prior probation experience outside Federal system & hold sanctions-based (non-incentives) philosophy toward participants. Will use CM in court process.	No specialized court. Has behavioral modification program run by PO but using CM with general supervision. Chief & POs experienced w/EBP & receptive to CM as EBP.	Frontline PO working with halfway house will implement CM. Halfway House protocol is sanctions–focused; PO wants to keep that focus even with CM.

Key

AUSA: Assistant United States Attorney (prosecutor).

FPD: Federal Public Defender (defense attorney).

PO: Probation Officers.

n = JSTEPS Team Members Per Site.

*Table from Rudes et al. (Rudes et al. 2011).

### Using PDSA to implement the CM protocol

In each probation agency, the PDSA process guides users through several stages of organizational change emphasizing exposure, understanding, adoption, adaptation, and implementation. Knowing that innovations that align with existing organizational cultures and practices generally see broader success (Knudsen et al. 2004), JSTEPS uses a continual and iterative process framed by Rogers diffusion theory to consider if and how a CM rewards-based system suits a community corrections environment (Rogers 1995). The JSTEPS PDSA process helps justice agencies determine whether the CM innovation is: 1) relatively advantageous over current practice; 2) in-line with current organizational mission, goals, values, and/or practices (compatibility); 3) consistent with previous ideas and concepts (compatibility; some complexity in terms of some of the required steps to implement use a CM protocol in standard probation sessions); 4) developable into operational practice (trialability), and 5) “felt” by organizational members (observability).

#### Study protocol: plan-Do-study-Act to implement CM through JSTEPS

The JSTEPS intervention facilitates site progress through CM design sessions and ongoing “revise and refine” learning collaborative discussions, and a project-specific software program. This iterative process allows both researchers and sites to learn from each other throughout the implementation process.

#### Learning session 1: developing each Site’s CM protocol

The first PDSA meeting focuses on the core principles of the EBP. Research sites receive expert training on CM as an EBP. This training includes information about findings from major studies, different approaches to developing point schemes, different types of rewards that may work, and the integration of sanctions with specific attention to prior work on the importance of swift, certain and fair sanctions in criminal justice processing. Specifically, the first learning session facilitates an appreciation for and understanding of CM principles with an emphasis on the factors that are important for implementing a science-based CM process within site-specific contexts. As these agencies and courts represent complex organizational structures, the study team makes considerable effort to include all team members at both collaborative learning sessions. Table 1 shows that in the JSTEPS study four of the five courts sent a multi-disciplinary team to the training, while one sent only probation supervisors and staff.

At this first learning session, researchers ask teams to consider the following CM principles in developing their protocol: 1) provide positive incentives to clients via a point system; 2) establish clear guidelines about required and point-earning behaviors; 3) emphasize abstinence as a key objective; 4) provide adequate incentives early in the program to get clients started off on the right foot; 5) use point escalation to promote sustained good performance; 6) integrate the point system into the agency’s normal operations; 7) use point bonuses to reinforce incentives for positive behavior, and 8) contract for no more than three behaviors at a time. The research team assembled these principles from limited previous research on using CM within criminal justice settings (Friedmann et al. 2008) including a review of the CM literature and recommendations from Dr. Maxine Stitzer, a CM expert. Researchers encouraged JSTEPS study sites to design CM protocols that align with their current systems, practices and policies while also considering the eight CM principles.

To facilitate the learning collaborative meeting, the research team developed a CM manual (Taxman et al. 2010) complete with guidelines on developing CM protocols within justice settings and information pertaining to: 1) the science behind CM; 2) point schemes (ways of standardizing a CM protocol for implementation); 3) diverse rewarding schemes (examples of rewards to incentivize participants); 4) sanctioning within a CM protocol; and 5) organizational action strategies. Each manual section also includes a series of worksheets for interactive exercises addressing the CM learning process. Finally, the manual contains exercises to assist the JSTEPS site teams with understanding each component of the CM process.

#### Post-learning session #1 activities

After the first learning session, learning teams have the charge to develop their CM protocol and point system. During this time, communication between the research team and collaborative team is maintained. A technical consultant continues routine quarterly phone calls with each team to discuss their progress and to field questions. The research team prepares feedback reports for each site based on a review of their draft CM protocols. The report addresses the degree to which the CM plans are consistent with the CM principles. This feedback loop within the PDSA process provides researchers with objective analysis of the plans and an opportunity to address discrepancies between a stated emphasis on evidence-based interventions and each sites’ existing “way of doing business.” Feedback reports allow the sites to compare and map their planned CM protocol against the CM science.

**Figure 2:**
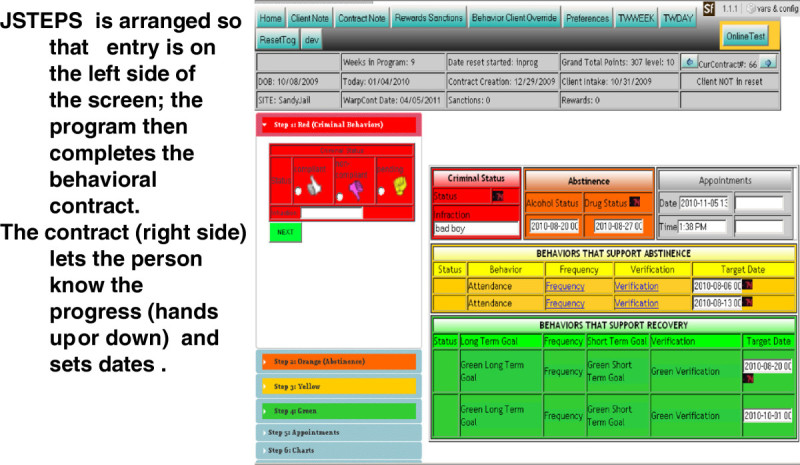
**JSTEPS software screenshot.**

Figure 2 is an example of a feedback report. The feedback report emphasizes the eight core CM principles and compares site protocols to these principles. It also uses the JSTEPS software (described below) to hypothetically demonstrate how each sites’ point system would work, noting when study subjects (offenders) would receive rewards. Additionally, the report asks sites to consider whether their self-designed CM protocol: 1) emphasizes core positive reinforcement principles; 2) gives offenders early rewards; and 3) requires any changes before they begin protocol implementation. These factors are considered important as part of the science of behavior change. (For a complete description of JSTEPS point and reward systems refer to Rudes and colleagues (Rudes et al. 2011)).

#### Continued support, training, feedback & analysis

As part of the PDSA process, each site receives on-going support, training and feedback via on-site, phone and email-based technical assistance (TA) and written reports provided by the research team. The focus of these quarterly sessions is to provide additional and on-going feedback on the use of CM. The research team produces a standardized report for each site that illustrates the number of clients using the protocol, the number of accumulated points, the number of days from starting CM to obtaining the first reward, the average points per client in the system, and general client outcomes. Qualitative researchers participate in these TA calls to learn about issues sites are experiencing, to document how actors understand CM and JSTEPS, and to understand how they are working CM into their routines and practices.

#### JSTEPS software

**Figure 3:**
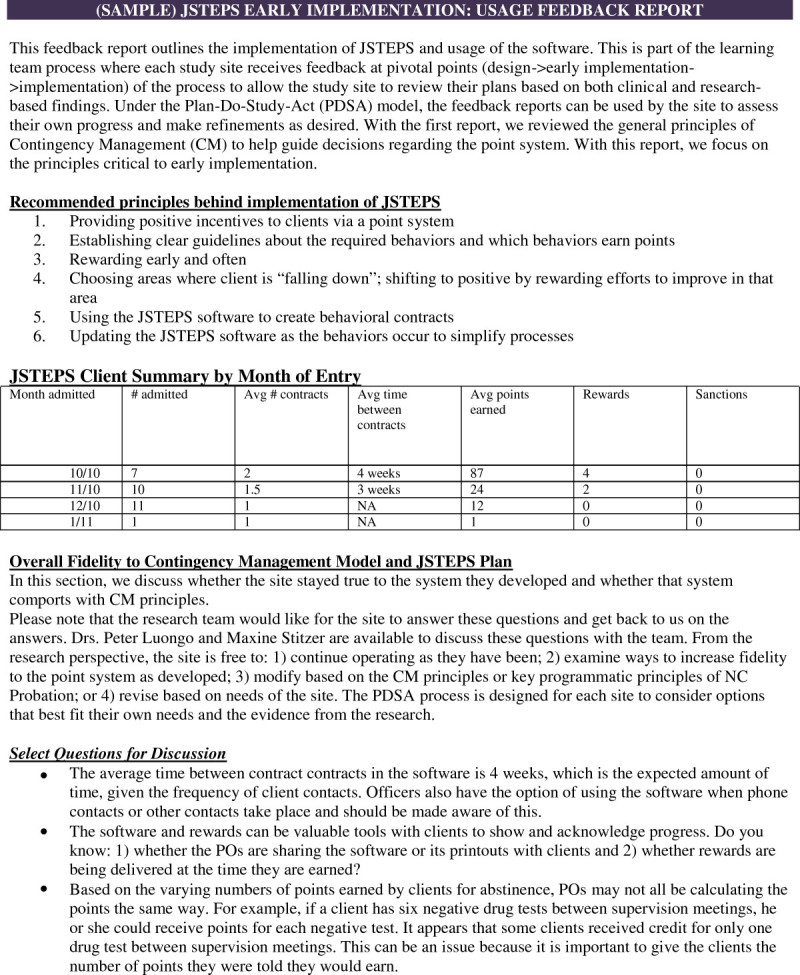
**Sample feedback report.**

The JSTEPS’ web-based software program houses each site-specific CM protocol. Sites receive the software and accompanying training after they participate in the first learning session and design a CM point and reward system for their jurisdiction. The JSTEPS software program is updateable at each contact to allow initiation and renewal of behavioral contracts (written CM protocol agreements) and distribution of points and rewards. A software manual (http://www.jsteps.org) facilitates the use of the tool (Taxman et al. 2010). Criminal justice actors use the program to enroll offenders in the CM protocol, provide updates on their progress (with the points built in to avoid manual means of keeping tabulations of the earned points), and create progress reports on the point accumulation for each target behavior. POs log data regarding probationer-specific goals in up to four color-coded areas tracking: criminal behaviors (red), drug testing/results behaviors (orange), attendance (at treatment, court, with PO) behaviors (yellow) and pro-social behaviors such as getting job or a driver’s license (green). The JSTEPS software keeps track of point tallies and alerts POs when rewards are due. The progress reports are useful for monitoring the impact of CM on behavior and helping POs illustrate probationers’ steps towards meeting desired behavioral goals. The data from the software or chart reviews are used to conduct fidelity checks on the use of CM and rewards. The data is used to measure number and type of positive behaviors, use of rewards, timing of rewards (length of time to obtain points and awards), use of sanctions, and timing of sanctions. These measures are used to assess the degree to which CM was implemented. Feedback reports (on the quarterly basis) provided incremental feedback to study sites on adherence to their CM schedules. Figure 3 shows a graphic depiction of the software.

#### Learning session #2: reviewing CM in each jurisdiction

Approximately 15 months after the first learning collaborative, the JSTEPS team hosts a second learning collaborative for all site teams. JSTEPS study participants assemble at a two-day meeting to review CM-related progress and plans and receive continued researcher and TA feedback. As part of the process, each study site gives a ten-minute presentation that reviews their sites’ progress and challenges in implementing CM. Researchers provide each site with a PowerPoint slide template to complete to ensure systematic presentation and data collection. Sites make presentations on five topic areas including: 1) description of CM; 2) site adherence to CM principles; 3) software and process challenges; 4) positive process or outcome experiences; and 5) data on how enrolled participants felt about CM. Sites also participate in a series of interactive working groups aimed at encouraging self-assessment and CM adherence.

### Data collection and measures

#### Administrative surveys

The JSTEPS study includes an organizational survey to understand the opinions and attitudes of justice actors in the study sites at the beginning of the study (baseline) and 24 months later. The survey includes two sections. Section One poses questions regarding: 1) the characteristics of the District Court respondents’ attitudes toward rehabilitation and punishment (Taxman et al. 2007b); 2) the degree of inter- and intra-agency collaboration (Fletcher et al. 2009); and 3) their attitudes toward EBPs and incentives. In this section, there are basic demographics such as the respondents’ level of education, area of concentration in education, position, social demographics, years of experience, and other fields in which respondents have worked. Section Two of the survey addresses the operations of the specialty courts and solicits opinions regarding CM components.

The baseline JSTEPS organizational survey draws from a number of sources. A major component was Kirby’s Provider Survey of Incentives (PSI), a 44-item instrument designed to solicit provider opinions on incentives (Kirby et al. 2006). Researchers hypothesize that CM is less widespread because of its cost, associated workload, difficulty of implementation, lack of fit with current interventions, and philosophical objections (Kirby et al. 1998; McGovern et al. 2004; Petry & Simcic 2002). Since the JSTEPS sample consists primarily of criminal justice personnel, the study team modified the PSI for use with this population (Murphy et al. 2012).

#### Client-level data

The study also collects client-level data to monitor the use of the CM and understand basic supervision outcomes. The JSTEPS software documents the behaviors of interest for each client and the points earned at each probation or problem-solving court meeting. Sites that do not use the software provide logs of behaviors and points earned. Another source of data is the administrative data used by the probation office including: client characteristics (e.g., age, gender, criminal history, criminal justice offense, type of sentence, etc.), progress on probation (e.g., number of probation visits, types of conditions, drug use, treatment completion, etc.), and outcomes (e.g., new arrests, probation violations and revocations, completion, etc.). These data support analyses to examine how different CM approaches affect client outcomes. The research team also uses administrative data to construct a comparison group of offenders that are similar to the CM group (on basic demographic characteristics) at each site to assess the degree to which the CM protocol influences offender outcomes.

#### Initial site visits

Two qualitative researchers travel to all five sites to interview and observe staff. During visits, researchers conduct semi-structured interviews with all team members including POs, judges, prosecutors and defense attorneys on five key themes including: 1) pre-study understandings of the role of probation in the justice process; 2) personal/organizational philosophies; 3) workplace routines; 4) inter- and intra-organizational collaborations; and 5) present knowledge of CM and/or behavioral modification strategies in correctional environments. The researchers use ethnographic techniques, asking questions as they naturally occurred in conversations to build rapport with subjects and to increase the depth of information gathered (Emerson 2001). Researchers address all key themes at all sites.

#### Follow-up site visits

One year after the first learning collaborative, qualitative researchers conduct a second set of visits to each of the five sites, and again focus on the same five themes. Additionally, researchers include a sixth theme specifically considering perceptions and use of CM in each site as it relates to organizational context, culture and environment. A final follow-up site visit occurs after 24-months of implementation to review the progress made at each site and to increase understanding regarding how contextual issues affect the use of CM in probation settings.

#### Overall data yield and sources

Quantitative data collection for the JSTEPS project includes baseline surveys and collection of data from the JSTEPS software and the U.S. Probation’s internal data management software (PACTS). Survey data provide information pertaining to acceptability and feasibility of implementing CM into justice settings (outcome), while client data allow researchers to see which types of clients are most likely to enroll and succeed in JSTEPS.

Qualitative data collection for this project reflects both breadth and depth throughout the project’s tenure. Qualitative researchers use every possible opportunity to collect data from research sites and subjects. Altogether, the qualitative data set will include: 1) written field notes from every site visit, learning collaborative session and other meetings totaling nearly 400 hours over three years; 2) typed verbatim transcripts from telephone calls with site participants; 3) researcher-derived feedback reports to sites; 4) all email exchanges between study sites and any member of the JSTEPS team, and 5) participant-provided written and visual data from PowerPoint presentations at public meetings and learning collaborative sessions. Table 2 provides a data map that outlines data collection methods and potential yield throughout the project.

**Table 2:** **Data map – data yield via quant + qual methods**

Research methods	Data yield	
Quantitative	Outcome	Process
Survey of officers	Participant responses such as	
	Attitudes and opinions of rewards	
Client data		
-JSTEPS software	Client data—drug use	Data re: system use
	Completion of treatment	
-Internal organizational system	Client data—client completion of treatment and probation use of revocation	
**Qualitative**		
Observations	Client & Participant data	Fidelity, uptake, adaptation, perception, use, acceptability, feasibility implementation
Interviews	Client & Participant data	Fidelity, uptake, adaptation, perception, use, acceptability, feasibility implementation
Focus groups	Participant data	Fidelity, uptake, adaptation, perception, use, acceptability, feasibility implementation
Phone calls	Client & Participant data	Fidelity, uptake, adaptation, perception, use, acceptability, feasibility implementation
Emails	Client & Participant data	Fidelity, uptake, adaptation, perception, use, acceptability, feasibility implementation
Learning collaborative	Client & Participant data	Fidelity, uptake, adaptation, perception, use, acceptability, feasibility implementation
On-going presentations	Client & Participant data	Fidelity, uptake, adaptation, perception, use, acceptability, feasibility implementation

## Discussion

The JSTEPS project proceeds from the assumption that “all implementation is local,” and focuses on understanding the challenges and decision-making involved as probation agencies balance locally necessary adaptations of contingency management strategies with the need to adhere to the core components this evidence-based practice. The JSTEPS PDSA implementation process arms participating sites with information about contingency management and allows them to design and implement CM protocols that are responsive to local circumstances. Both quantitative and qualitative data collection strategies are used throughout the adoption and implementation phases to understand site decision-making processes and to further document the implementation process—a critical facet of the PDSA approach. Learning collaboratives and feedback reports give sites specific information about their own CM implementation and are structured to increase fidelity between site-specific adaptations and core CM principles. The goal is to sensitize sites to the major mechanisms behind CM that can facilitate client-level change. Together, the JSTEPS PDSA implementation processes allow sites to “act” or refine their CM protocols to adapt to the local context. Analysis of the longitudinal data using both quantitative and qualitative methods provides an opportunity to contribute to the discussion of the cycles of change—how sites move from adoption to implementation to penetration into core agency practices.

The JSTEPS study protocol provides a unique opportunity to learn about the concept of transportability through a focus on integration of CM into justice settings. By having each site design and implement their own version of CM that fits the sociopolitical culture of the organization and setting, researchers obtain a better appreciation for the lifecycle of implementation. In theory, the emphasis on local adaptations within a set of core parameters should enhance the likelihood of long-term sustainability of CM in these settings over time. Even more important, the process provides a unique opportunity to observe how practitioners adapt an evidence-based practice while still being mindful of its core scientific principles. The focus on transportability provides a novel contribution to understanding setting, intervention, environment, and decision-making processes that teams of professionals encounter as part of an implementation process. The value of the study lies in documenting and understanding the trials and tribulations that each site will encounter as they continue to learn how to shape the CM intervention to suit their environment.

## Authors’ information

Faye S. Taxman is University Professor, George Mason University, Department of Criminology, Law & Society; and Director of the Center for Advancing Correctional Excellence (ACE!). Danielle Rudes is Assistant Professor, George Mason University, Department of Criminology, Law & Society; and Deputy Director of the Center for Advancing Correctional Excellence (ACE!).

## Authors’ original submitted files for images

Below are the links to the authors’ original submitted files for images. Authors’ original file for figure 1 Authors’ original file for figure 2 Authors’ original file for figure 3
