# Supplementary material for: Implementation of contingency management in probation agencies using a case controlled longitudinal design: a PDSA study protocol
Source: Health Justice. 2013 Dec 19;1:7. doi: 10.1186/2194-7899-1-7 (PMC5120660; doi:10.1186/2194-7899-1-7)
Supplement: Supplementary file 1 — Authors’ original file for figure 1 [file 40352_2013_4_MOESM1_ESM.pdf]

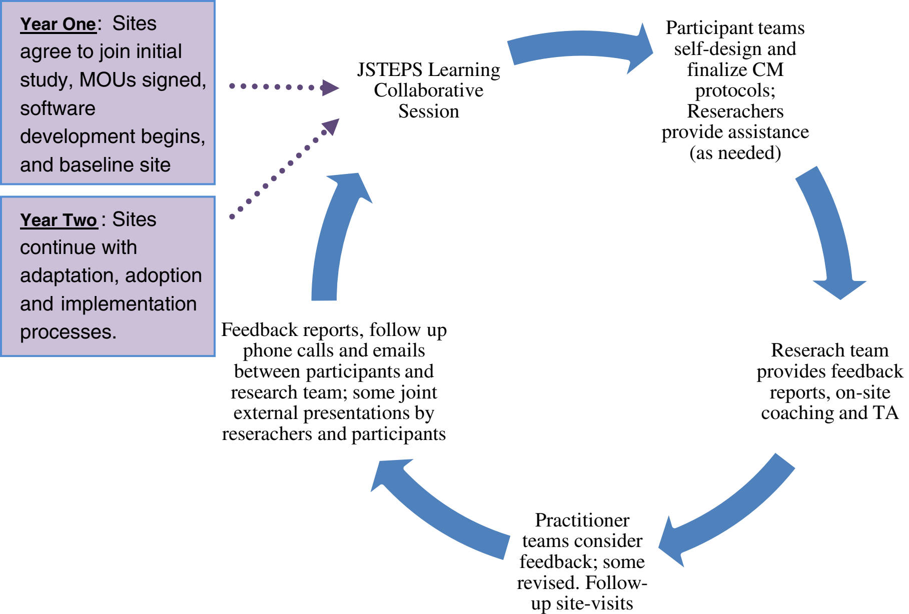
Year One: Sites agree to join initial study, MOUs signed, software development begins, and baseline site
Year Two : Sites continue with adaptation, adoption and implementation processes.
JSTEPS Learning Collaborative Session
Participant teams self-design and finalize CM protocols; Reserachers provide assistance (as needed)
Reserach team provides feedback reports, on-site coaching and TA
Practitioner teams consider feedback; some revised. Follow-up site-visits
Feedback reports, follow up phone calls and emails between participants and research team; some joint external presentations by reserachers and participants
