# Supplementary material for: Implementation of contingency management in probation agencies using a case controlled longitudinal design: a PDSA study protocol
Source: Health Justice. 2013 Dec 19;1:7. doi: 10.1186/2194-7899-1-7 (PMC5120660; doi:10.1186/2194-7899-1-7)

JSTEPS is arranged so that entry is on the left side of the screen; the program then completes the behavioral contract.

The contract (right side) lets the person know the progress (hands up or down) and sets dates .

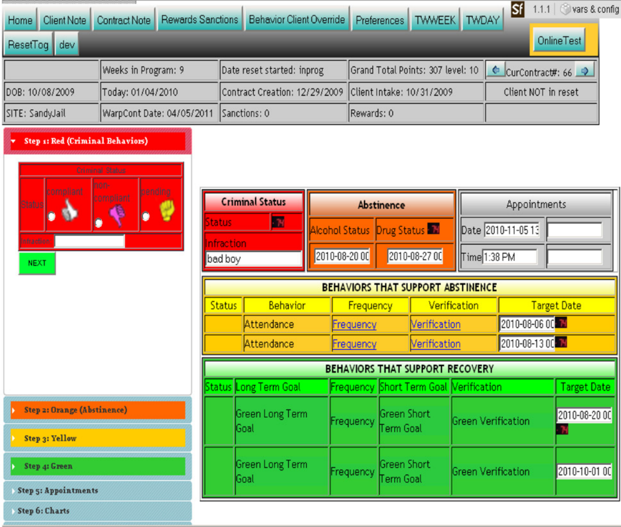

Supplement: Supplementary file 2 — Authors’ original file for figure 2 [file 40352_2013_4_MOESM2_ESM.pdf]
