# Supplementary material for: Implementation of contingency management in probation agencies using a case controlled longitudinal design: a PDSA study protocol
Source: Health Justice. 2013 Dec 19;1:7. doi: 10.1186/2194-7899-1-7 (PMC5120660; doi:10.1186/2194-7899-1-7)
Supplement: Supplementary file 3 — Authors’ original file for figure 3 [file 40352_2013_4_MOESM3_ESM.pdf]

(SAMPLE) JSTEPS EARLY IMPLEMENTATION: USAGE FEEDBACK REPORT

This feedback report outlines the implementation of JSTEPS and usage of the software. This is part of the learning team process where each study site receives feedback at pivotal points (design->early implementation->implementation) of the process to allow the study site to review their plans based on both clinical and research-based findings. Under the Plan-Do-Study-Act (PDSA) model, the feedback reports can be used by the site to assess their own progress and make refinements as desired. With the first report, we reviewed the general principles of Contingency Management (CM) to help guide decisions regarding the point system. With this report, we focus on the principles critical to early implementation.

Recommended principles behind implementation of JSTEPS

1. Providing positive incentives to clients via a point system
2. Establishing clear guidelines about the required behaviors and which behaviors earn points
3. Rewarding early and often
4. Choosing areas where client is "falling down"; shifting to positive by rewarding efforts to improve in that area
5. Using the JSTEPS software to create behavioral contracts
6. Updating the JSTEPS software as the behaviors occur to simplify processes

JSTEPS Client Summary by Month of Entry

Month admitted	# admitted	Avg # contracts	Avg time between contracts	Avg points earned	Rewards	Sanctions
10/10	7	2	4 weeks	87	4	0
11/10	10	1.5	3 weeks	24	2	0
12/10	11	1	NA	12	0	0
1/11	1	1	NA	1	0	0

Overall Fidelity to Contingency Management Model and JSTEPS Plan

In this section, we discuss whether the site stayed true to the system they developed and whether that system comports with CM principles.

Please note that the research team would like for the site to answer these questions and get back to us on the answers. Drs. Peter Luongo and Maxine Stitzer are available to discuss these questions with the team. From the research perspective, the site is free to: 1) continue operating as they have been; 2) examine ways to increase fidelity to the point system as developed; 3) modify based on the CM principles or key programmatic principles of NC Probation; or 4) revise based on needs of the site. The PDSA process is designed for each site to consider options that best fit their own needs and the evidence from the research.

Select Questions for Discussion

- The average time between contract contracts in the software is 4 weeks, which is the expected amount of time, given the frequency of client contacts. Officers also have the option of using the software when phone contacts or other contacts take place and should be made aware of this.
- The software and rewards can be valuable tools with clients to show and acknowledge progress. Do you know: 1) whether the POs are sharing the software or its printouts with clients and 2) whether rewards are being delivered at the time they are earned?
- Based on the varying numbers of points earned by clients for abstinence, POs may not all be calculating the points the same way. For example, if a client has six negative drug tests between supervision meetings, he or she could receive points for each negative test. It appears that some clients received credit for only one drug test between supervision meetings. This can be an issue because it is important to give the clients the number of points they were told they would earn.
